# An inclined detector geometry for improved X-ray total scattering measurements

**DOI:** 10.1107/S1600576723001747

**Published:** 2023-04-01

**Authors:** Nicholas Burns, Aly Rahemtulla, Scott Annett, Beatriz Moreno, Stefan Kycia

**Affiliations:** a Department of Physics, University of Guelph, Guelph, Ontario, Canada; b Canadian Light Source, Saskatoon, Saskatchewan, Canada; Montanuniversität Leoben, Austria

**Keywords:** synchrotron radiation, X-ray diffraction, total scattering, instrumentation, area detectors

## Abstract

X-ray total scattering measurements are implemented using a digital flat-panel area detector in an inclined geometry and compared with the traditional geometry. The traditional geometry is defined here by the incident X-ray beam impinging on and normal to the center-most pixel of a detector. The inclined geometry is defined here by a detector at a pitch angle α, set to 15° in this case, bisected by the vertical scattering plane. The detector is positioned such that the incident X-ray beam strikes the pixels along the bottom edge and 90° scattered X-rays impinge on the pixels along the top edge. The geometric attributes of the inclined geometry translate into multiple benefits, such as an extension of the measurable scattering range to 90°, a 47% increase in the accessible magnitudes of the reciprocal-space vector *Q* and a leveling of the dynamic range in the measured total scattering pattern. As a result, a sixfold improvement in signal-to-noise ratios is observed at higher scattering angles, enabling up to a 36-fold reduction in acquisition time. Additionally, the extent of applied modification functions is reduced, decreasing the magnitude of termination ripples and improving the real-space resolution of the pair distribution function *G*(*r*). Taken all together, these factors indicate that the inclined geometry produces higher quality data than the traditional geometry, usable for simultaneous Rietveld refinement and total scattering studies.

## Abstract 2

An inclined geometry is investigated for X-ray total scattering measurements using a digital flat-panel area detector. The inclined geometry enables acquisition of higher quality data for simultaneous Rietveld refinement and total scattering studies, yielding structural information on the short-, medium- and long-range orders from one single measurement.

## 1. Introduction

The reduced pair *G*(*r*) and radial *R*(*r*) distribution functions have been instrumental in characterizing condensed matter systems by means of X-ray total scattering experiments (Billinge, 2019 ▸; Petkov, 2012 ▸). The distribution function methodology has been successful in analyzing large subsets of materials such as melts and amorphous and polycrystalline systems (Laaziri *et al.*, 1999*a* ▸,*b* ▸; Petkov *et al.*, 1999 ▸; Skinner *et al.*, 2013 ▸; Tomberli *et al.*, 2015 ▸). In order to produce a high real-space resolution *G*(*r*) by means of a Fourier transform, the measurement of the X-ray total scattering pattern’s coherent component should be taken out to large values in *Q*, the magnitude of the reciprocal-space vector. Here, *Q* is defined in equation (1), where λ is the wavelength of the incident X-ray beam and 2θ is the scattering angle,




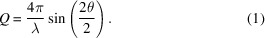

Increasingly, effort is being dedicated to reducing image acquisition times without compromising real-space resolution in *G*(*r*). The push is fueled by challenging experiments in thin films, systems of low atomic number and time-resolved *in situ* measurements (Jensen *et al.*, 2015 ▸; Li *et al.*, 2021 ▸; Wiaderek *et al.*, 2013 ▸; Chapman *et al.*, 2015 ▸).

**Figure 1:**
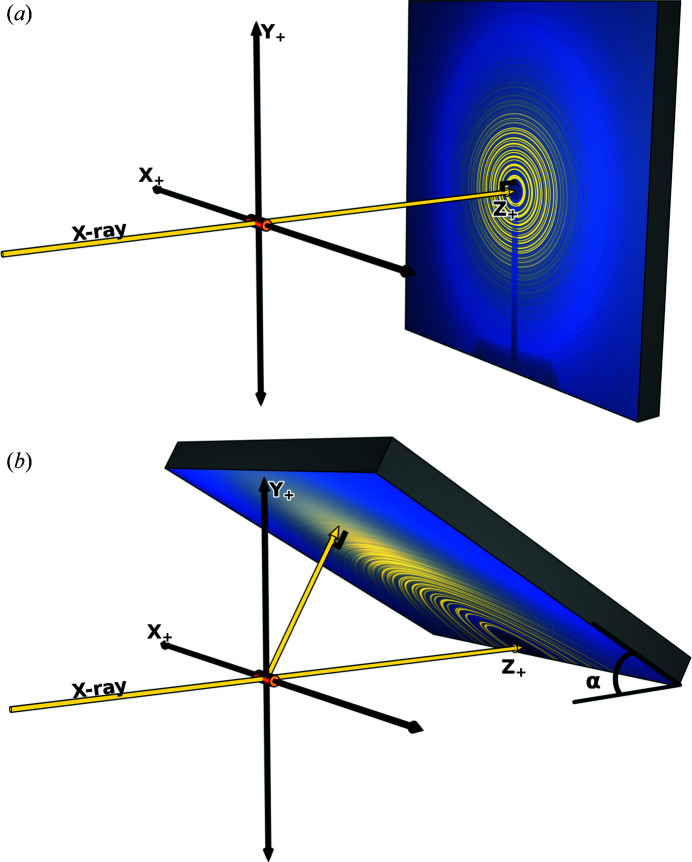
(*a*) The traditional detector geometry: the incident X-ray beam impinges on and is normal to the center-most pixel of the detector. (*b*) The inclined detector geometry: the detector is at a pitch angle α of 15°, bisected by the vertical scattering plane. The detector is positioned such that the incident X-ray beam strikes the pixels along the bottom edge and 90° scattered X-rays impinge on the pixels along the top edge.

In order to achieve the desired counting statistics and fast acquisition times, two-dimensional digital flat-panel area detectors are commonly used when performing X-ray total scattering measurements (Marlton *et al.*, 2019 ▸). Traditional detector geometries have the incident X-ray beam normal to the detector surface, as shown in Fig. 1 ▸(*a*). In these geometries, the experimenter must find a balance between the X-ray beam energy, the sample-to-detector distance (SDD) and the quality of the acquired measurement. Increasing the X-ray energy extends the maximum reachable value of *Q*, while conversely decreasing the measured scattered intensity by reducing the incident X-ray beam flux and reducing the interaction cross sections for both the sample and detector (Waseda, 2003 ▸; Hubbell & Seltzer, 1995 ▸). Decreasing the SDD extends the measurable scattering range but shifts the dynamic range of the measured X-ray total scattering pattern by means of the solid angle, which becomes larger at lower scattering angles and smaller at higher scattering angles. This results in possible detector saturation at lower scattering angles and poor signal-to-noise ratios at higher scattering angles. In addition, decreasing the SDD in the traditional geometry leads to compromises in the quality of the measured lower scattering angle region, as the resolution of *Q* is decreased and the beam-stop shadow blocks more of the lower angle scattering pattern.

In order to address many of these issues, we explore here the performance of an inclined detector geometry. The inclined geometry is defined by a detector at a pitch angle α, set to 15° in this case, bisected by the vertical scattering plane. The detector is positioned such that the incident X-ray beam strikes the pixels along the bottom edge and 90° scattered X-rays impinge on the pixels along the top edge, as shown in Fig. 1 ▸(*b*). Lower pitch angles maximize the favorable attributes of the geometry. However, collisions between the detector and the sample rotation stage prevent lower pitch angles from being obtained. As a result, the minimum physically allowable pitch angle angle of 15° was chosen for this example. A series of synchrotron-based measurements were conducted in each configuration which prove the advantages of the inclined geometry over the traditional geometry. This was done by measuring a powdered silicon laboratory standard to calibrate the detector and a powdered nickel sample to serve as an unknown for comparison between the geometries. It was shown that, with careful two-dimensional image analysis, parallax corrections and addressing of intrinsic detector curvature, the inclined geometry is superior to the traditional geometry, producing higher quality data usable for simultaneous Rietveld refinement and total scattering studies.

## 2. Methods

X-ray total scattering patterns were collected for a powdered laboratory standard of silicon (NIST SRM 640f) and for powdered nickel (Sigma–Aldrich). All samples were measured at a temperature of 100 K for a summed total of 512 1 s exposures. The measurements were conducted on the high energy wiggler X-ray diffraction and scattering beamline in the Brockhouse sector of the Canadian Light Source (Gomez *et al.*, 2018 ▸). All measurements were performed with 60 keV X-rays. A PerkinElmer XRD 1621 CN3-EHS (PE-1621) flat-panel area detector containing 2048 × 2048 pixels with a pitch of 200 µm was used to measure two-dimensional X-ray total scattering patterns.

The flat-panel area detector was placed in two different geometries. In the first configuration, represented in Fig. 1 ▸(*a*), the detector was placed in the traditional geometry, defined with the incident X-ray beam impinging on and normal to the center-most pixel of the detector. In the second configuration represented in Fig. 1 ▸(*b*), the detector was placed in an inclined geometry, defined with the detector at a pitch angle α of 15°, bisected by the vertical scattering plane. The detector is positioned such that the incident X-ray beam strikes the pixels along the bottom edge and 90° scattered X-rays impinge on the pixels along the top edge. To avoid the loss of coherent intensity at higher scattering angles as a result of 98% horizontally polarized X-rays, the detector is strategically positioned to be bisected by the vertical scattering plane.

The PE-1621 is a commonly used detector on many beamlines for its large active area and relatively low cost. However, the PE-1621 introduces significant artifacts and distortions into the collected X-ray scattering patterns as a result of its electronic and physical design. Due to the electronic design, the detector exhibits a characteristic intensity distortion in the measured X-ray total scattering pattern. The intensity distortion is produced from a variation in read-out gain between pixels in the detector (Skinner *et al.*, 2012 ▸). The gain of each pixel is calculated through a flood field measurement, producing a correction for this PE-1621 detector which reduces the intensity distortion to an unobservable level (Burns, 2022 ▸). The physical design of the detector introduces parallax and topography distortions in the measured position of an X-ray beam incident on the flat-panel X-ray detector (Weiß *et al.*, 2012 ▸; Lüthi *et al.*, 2019 ▸; Wu *et al.*, 2002 ▸; Zaleski *et al.*, 1998 ▸). Using only the measured X-ray total scattering pattern of a powdered silicon laboratory standard at 100 K, these distortions were quantified and corrected. For detailed information on the parallax and topography distortion corrections in this work, see the supporting information. The corrections calculated from the silicon were applied to all measured X-ray total scattering patterns and in all geometries used in these experiments.

**Figure 2:**
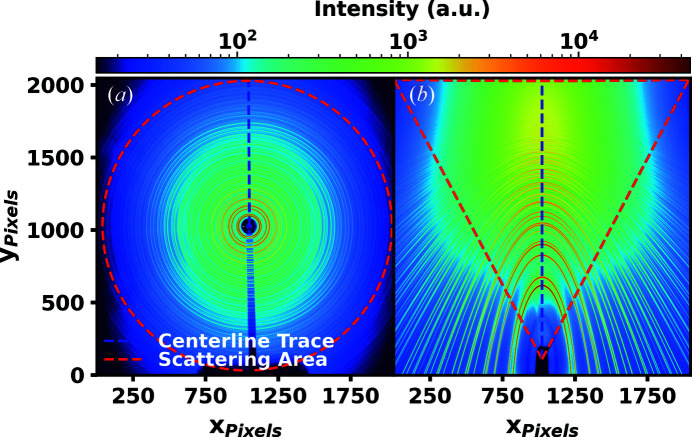
Selected scattering areas for powdered nickel. (*a*) The traditional geometry, which covers a full circular 360° in χ and extends to a maximum *Q* of 29 Å^−1^. The beam-stop shadow is masked and removed from any integrations. (*b*) The inclined geometry, covering a 125° wedge in χ bisected by the vertical scattering plane and extending to a maximum *Q* of 42.5 Å^−1^.

The selected scattering areas used for generation of the distribution functions cover a different range in each geometry, as displayed in Fig. 2 ▸. In the traditional geometry, the selected scattering area covers a full circular 360° in the azimuthal angle χ, where the beam-stop shadow is masked and removed from any integrations. The maximum *Q* reached is 29 Å^−1^. In the inclined geometry, the selected scattering area covers a 125° wedge in χ bisected by the vertical scattering plane and a maximum *Q* of 42.5 Å^−1^ is reached. In the inclined geometry, the choice of a scattering area covering a smaller range in χ about the bisecting vertical scattering plane reduces peak broadening due to the scattering footprint of a line beam on the sample compared with the full circular χ range used in the traditional geometry (Sulyanov *et al.*, 1994 ▸; He, 2018 ▸). The same χ range could be chosen for the traditional geometry to make this effect equivalent in both cases but this would result in reduced signal-to-noise ratios.

## 3. Results and discussion

**Figure 3:**
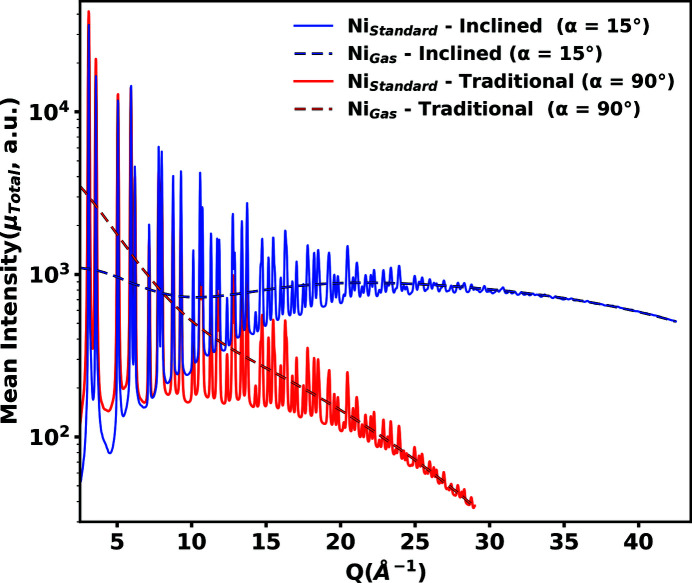
Total scattering intensity after background subtractions μ_Total_ in traditional and inclined geometries for experimentally measured powdered nickel (solid lines) and theoretically calculated nickel gas (dashed lines), integrated over the selected scattering areas for each geometry as defined in Fig. 2 ▸. Theoretical nickel gas intensities are calculated assuming no structure and the same density as the experimental nickel powder. The dynamic range of μ_Total_ in the inclined geometry is more level than that in the traditional geometry.

Implementation of the inclined geometry extends the accessible maximum scattering range to 90° and thus results in a 47% larger *Q* range. Additionally, the dynamic range of the measured X-ray total scattering pattern is leveled. The measured total scattering intensity of the lower scattering angle region is reduced, while the intensity of the higher scattering angle region is increased. Reducing the counts per pixel in the lower scattering angle region allows for the incident beam flux to be increased when measuring samples which saturate the detector in these lower regions. In this way, the scattered intensity of the entire pattern is increased, which results in an improved signal-to-noise ratio. In each experiment, the incident beam flux is adjusted such that the most intense pixels of the first significant Debye–Scherrer ring are measured at 45 000 counts. This is below the 65 000 count saturation limit of the detector. The inclined detector geometry is compared with the traditional by integrating along χ to a one-dimensional plot in *Q*, within the scattering areas as defined in Fig. 2 ▸. Interpolation and integration from the collected two-dimensional scattering patterns to one-dimensional patterns in *Q* are performed using custom Python code which allows for simultaneous correction of the parallax and topography distortions. For detailed information on the parallax and topography distortion corrections used in this work, see the supporting information. Fig. 3 ▸ shows the integrated mean total scattering intensities after background subtractions μ_Total_ for powdered nickel in both geometries.

The solid angle subtended by a pixel ΔΩ(γ) is quantified using equation (2), where *P*_*x*,*y*_ is the pixel pitch, γ is the incidence angle and SDD is the sample-to-detector distance (Grillo, 2008 ▸),

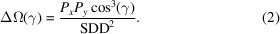

**Figure 4:**
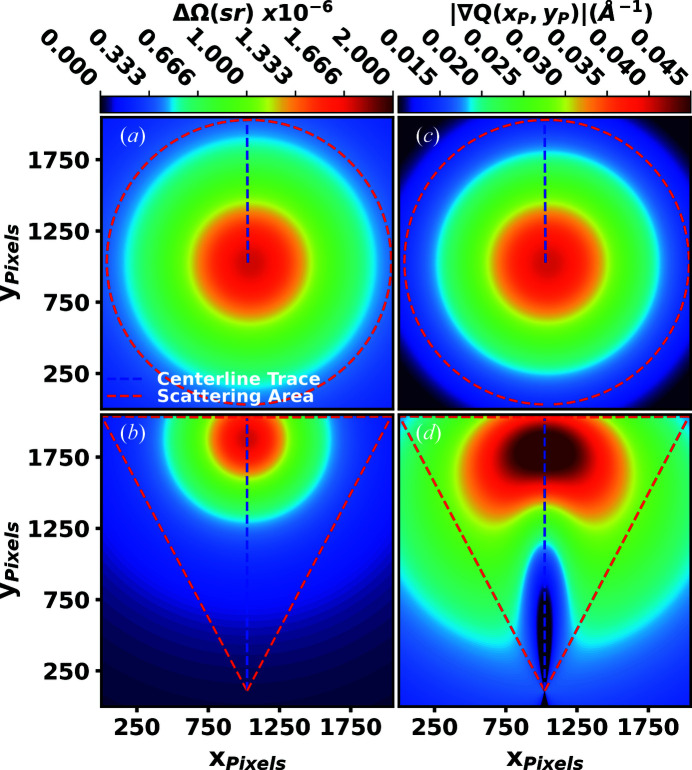
The solid angle subtended by a pixel ΔΩ(γ) is presented in (*a*) the traditional geometry and (*b*) the inclined geometry. The magnitude of the reciprocal-space vector gradient |∇*Q*(*x*_P_, *y*_P_)| is presented in (*c*) the traditional geometry and (*d*) the inclined geometry. Implementing the inclined geometry shifts the PONI position, changing ΔΩ(γ) and |∇*Q*(*x*_P_, *y*_P_)| such that they are reduced in the lower scattering angle regions and increased in the higher scattering angle regions.

The most significant geometric effect introduced by implementation of the inclined detector geometry is the change to the distribution of ΔΩ(γ) across the detector surface. The point of normal incidence (PONI) is the point of maximum ΔΩ(γ) for flat detectors of uniform pixel size. The PONI in the traditional geometry is positioned at a scattering angle 2θ = 0°. In the inclined geometry, the PONI is repositioned away from the beam center to the scattering angle 2θ = 90° − α along the vertical scattering plane. Shifting the PONI has the effect of decreasing ΔΩ(γ) in the lower scattering angle region, reducing the measured counts per pixel, and increasing ΔΩ(γ) in the higher scattering angle region, resulting in more measured counts per pixel. Figs. 4 ▸(*a*) and 4 ▸(*b*) show ΔΩ(γ) in the traditional and inclined geometries, respectively.

The solid angle indicates both the change in pixel intensity and the change in angular resolution of the pixel. However, when considering powder diffraction, the resolution between the pixels along the azimuthal direction is not typically relevant, as total scattering measurements are integrated along this dimension. In order to investigate only the resolution along the direction parallel to the scattering angle, the magnitude of the reciprocal-space vector gradient |∇*Q*(*x*_P_, *y*_P_)| is calculated using equation (3),

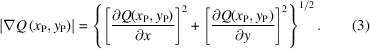

Here, *x*_P_ and *y*_P_ are the two pixel coordinates defined along the detector’s surface. |∇*Q*(*x*_P_, *y*_P_)| quantifies the amount of *Q* covered per pixel along the direction parallel to the scattering angle after multiplying by the pixel size, which is simply one in pixel space. The smaller the value of |∇*Q*(*x*_P_, *y*_P_)|, the more pixels will fit within the width of a measured Debye–Scherrer ring. Comparing the inclined geometry and the traditional as shown in Figs. 4 ▸(*c*) and 4 ▸(*d*), |∇*Q*(*x*_P_, *y*_P_)| is reduced in the lower scattering angle region but increased in the higher scattering angle region.

**Figure 5:**
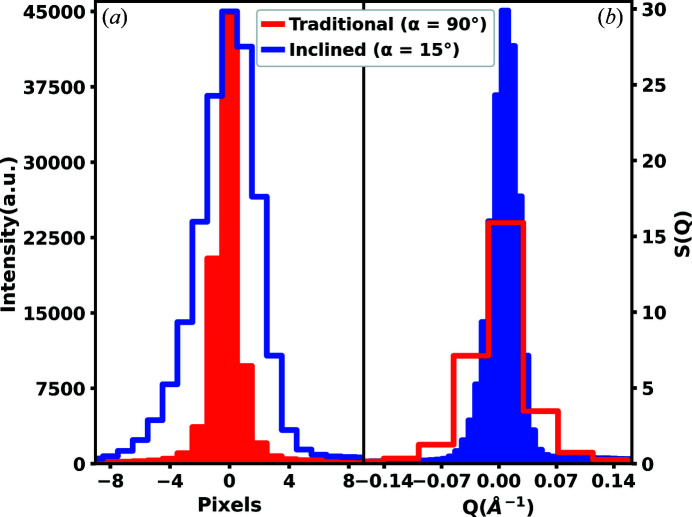
The first significant reflection of powdered nickel in traditional and inclined geometries for (*a*) the pixel intensity and (*b*) the structure function *S*(*Q*) of selected centerline traces as defined in Fig. 2 ▸. In both geometries, the incident beam flux is adjusted such that the most intense pixels in the first significant Debye–Scherrer ring are measured at 45 000 counts to avoid detector saturation. In the inclined geometry an advantageous trend is observed where the first significant reflection is broad in pixel space and sharp in *Q* space. The opposite disadvantageous trend is observed in the traditional geometry.

The geometric effect of changing |∇*Q*(*x*_P_, *y*_P_)| is highlighted in Fig. 5 ▸, by comparing the centerline traces as defined in Fig. 2 ▸, for the first significant Debye–Scherrer rings in both the traditional and inclined geometries. The centerline trace is plotted as intensity per pixel and in the structure function *S*(*Q*) as defined in equation (4), where *I*(*Q*) is the normalized isolated coherent intensity, *N* is the number of atoms illuminated by the incident X-ray beam and *f*(*Q*) is the coherent form factor for a monoatomic system (Peterson *et al.*, 2021 ▸),

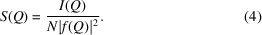




The benefits of a reduced |∇*Q*(*x*_P_, *y*_P_)| at lower scattering angles are also highlighted in Fig. 5 ▸. In Fig. 5 ▸(*a*), the line shape of the measured Debye–Scherrer ring in the inclined geometry appears broader in pixel space relative to the traditional geometry. However, it is important to understand that this broadening in pixel space does not translate to broadening in *Q* space, as shown in Fig. 5 ▸(*b*) for the first significant peak in *S*(*Q*). Here, the *Q*-space resolution is instead improved, as shown by a sharper and more highly sampled peak. The opposite is true for the traditional geometry. The inclined geometry case is preferred for X-ray total scattering measurements and analysis. Specifically, high *Q*-space resolution for intense low scattering angle Debye–Scherrer rings improves the real-space resolution envelope for generated *G*(*r*) functions as higher frequency signals may be captured. Additionally, increased sampling of the lower scattering angle region benefits traditional analysis methods for polycrystalline materials such as Rietveld refinement, which focuses on these lower scattering angle regions.

**Figure 6:**
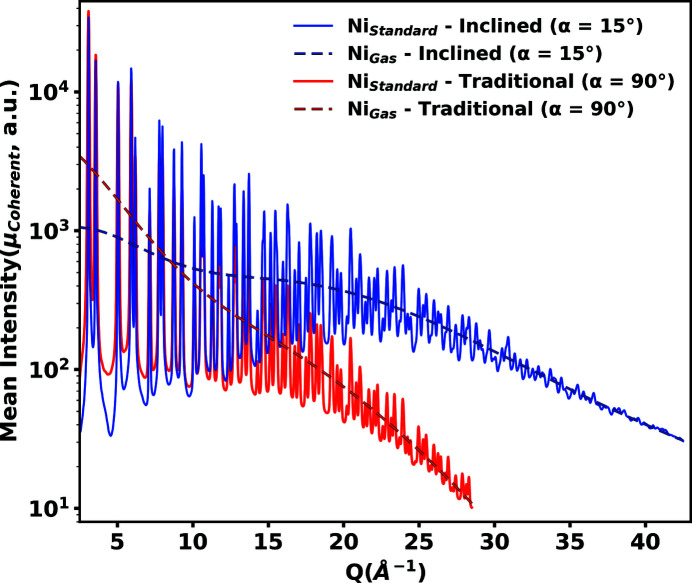
Unnormalized coherent scattering intensity μ_Coherent_ in traditional and inclined geometries for the experimentally measured powdered nickel (solid lines) and the theoretically calculated nickel gas (dashed lines), interpolated and integrated over the selected scattering areas for each geometry as defined in Fig. 2 ▸. Theoretical nickel gas intensities are calculated assuming no structure and the same density as the experimental nickel powder. Increased intensity in the higher scattering angle region is observed in the inclined geometry compared with the traditional geometry.

The theoretical incoherent and photoelectric intensities were subtracted out of μ_Total_ leaving only the integrated mean unnormalized coherent intensities μ_Coherent_ (Burns, 2022 ▸). We observed an increased μ_Coherent_ in the high scattering angle regions for the inclined geometry compared with the traditional geometry. A higher coherent intensity largely contributes to improved signal-to-noise ratios for the inclined geometry over the traditional geometry. A comparison of μ_Coherent_ is plotted in Fig. 6 ▸ for powdered nickel.

A comparison of the integrated mean unnormalized coherent intensity’s signal-to-noise ratio SNR_Coherent_ in the inclined and traditional geometries is calculated using experimentally determined Poisson-like statistics for the PE-1621 detector. The standard deviation 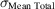 of an ensemble of binned snapshots and pixels for total scattering is given by equation (5),

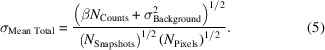

Here, *N*_Counts_ is the number of measured counts, *N*_Snapshots_ is the number of integrated snapshots, *N*_Pixels_ is the number of integrated pixels in χ, σ_Background_ is the standard deviation of the background after the subtraction of all of its signals and β is the conversion factor from visible photons to measured counts (Michel *et al.*, 2006 ▸; Hughes & Hase, 2010 ▸; Lyons, 2004 ▸). For detailed information on how the experimentally determined Poisson-like statistics were calculated for the PE-1621 detector, see the supporting information. SNR_Coherent_ can then be calculated from the ratio of μ_Coherent_ and 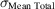 as defined in equation (6) (Lyons, 2004 ▸), 

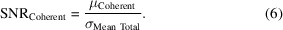

μ_Coherent_ is calculated by subtraction of the theoretically calculated incoherent and photoelectric signals from μ_Total_. As a result, the standard deviation of μ_Coherent_ remains as 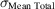 as it does not contain statistical noise and is assumed to be unchanged by the subtraction of the theoretical signal.

**Figure 7:**
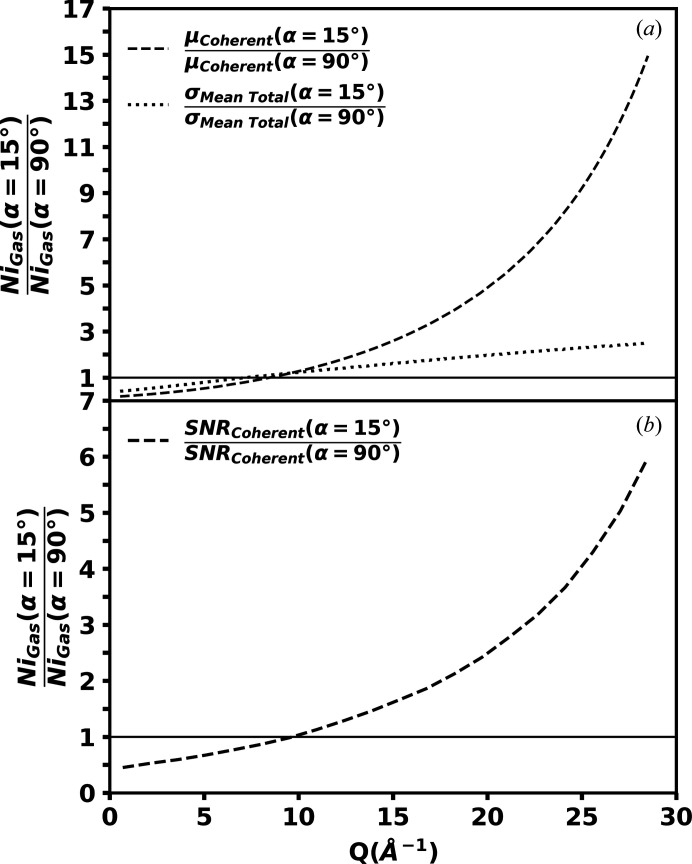
Ratio comparison of (*a*) the integrated mean unnormalized coherent intensity μ_Coherent_ and the standard deviation of the integrated mean total scattering intensity after background subtraction 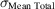 , and (*b*) the integrated mean unnormalized coherent intensity’s signal-to-noise ratio SNR_Coherent_ in the inclined geometry over that in the traditional geometry for theoretically calculated nickel gas as defined in Figs. 3 ▸ and 6 ▸. An increase in SNR_Coherent_ in the inclined geometry compared with the traditional geometry is observed for the higher scattering angle region, where SNR_Coherent_ is six times larger at the maximum *Q*, allowing for up to 36 times fewer snapshots to be acquired.

Ratio comparisons of μ_Coherent_ and 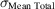 between the inclined and traditional geometries are shown in Fig. 7 ▸(*a*), calculated from the theoretical nickel gas intensities defined in Figs. 3 ▸ and 6 ▸. Experimentally determined values of β = 2.83 and σ_Background_ = 53 are used to calculate 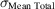 . Additionally, the ratio comparison of SNR_Coherent_ is shown in Fig. 7 ▸(*b*), from which it is observed that the SNR_Coherent_ of the inclined geometry is reduced in the lower scattering angle regions and increased in the higher scattering angle regions compared with that of the traditional geometry. This is an ideal compromise between the two geometries, as the signal-to-noise ratios are typically higher in the low scattering angle regions and lower in the high scattering angle regions. Effectively a trade-off in signal-to-noise ratio has been made, reducing SNR_coherent_ where it is not needed and increasing it where it is needed most. The ratio comparison of SNR_Coherent_ indicates that a measurement of the same quality up to 29 Å^−1^ could be acquired measuring 36 times fewer snapshots, greatly reducing the acquisition time.

The inclined geometry provides access to a broader range in *Q*. The additionally probed reciprocal space helps to preserve the real-space resolution by allowing the reduced total scattering structure function *F*(*Q*) defined in equation (7) to damp out naturally due to the Debye–Waller factor,

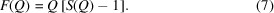

**Figure 8:**
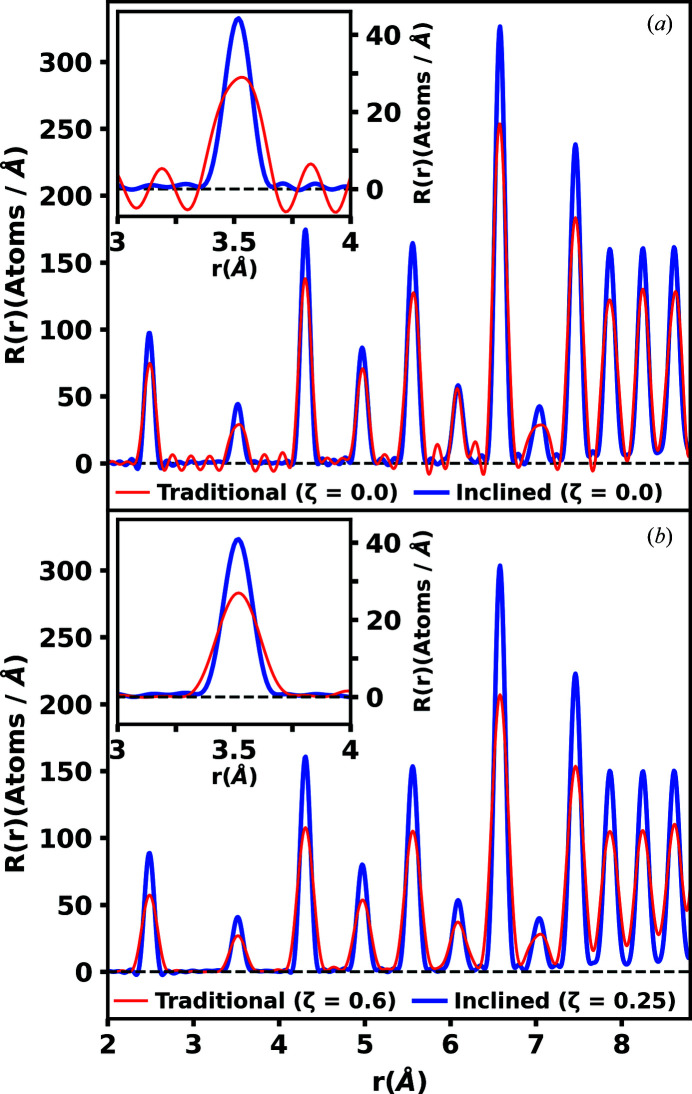
Radial distribution functions *R*(*r*) in inclined and traditional geometries for powdered nickel using (*a*) no modification function and (*b*) a Lorch power factor of ζ = 0.6 in the traditional geometry and ζ = 0.25 in the inclined geometry. The Lorch power factors were adjusted such that the magnitude of the termination ripples was reduced to a minimal and proportional level in both geometries. Greater real-space resolution and smaller termination ripples, which require less damping, are observed in the inclined geometry.

Modified Lorch functions with different Lorch power factors ζ were multiplied by the *F*(*Q*) functions in each geometry (Lorch, 1969 ▸). The modified *F*(*Q*) functions produce radial distribution functions *R*(*r*) containing minimal termination ripples of equivalent proportionality. The generated *R*(*r*) functions are compared in Fig. 8 ▸. The traditional geometry required a ζ value of 0.6, while the inclined geometry only required a ζ value of 0.25 in the case of the powdered nickel sample. For detailed information on the use of the modified Lorch function, see the supporting information.

**Figure 9:**
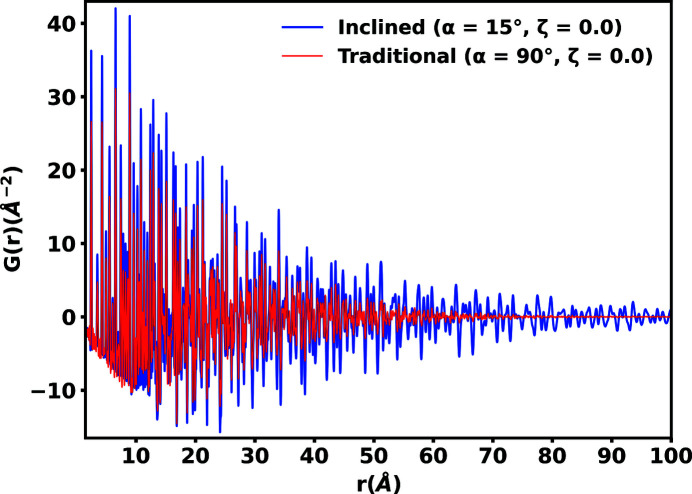
Pair distribution functions *G*(*r*) of powdered nickel in inclined and traditional geometries. No modification functions are applied in either geometry. Greater real-space resolution in the inclined geometry is observed, which extends past the point where the traditional geometry’s correlations are no longer observable.

Similar to *R*(*r*), the inclined geometry also produces high resolution *G*(*r*), as shown in Fig. 9 ▸. We observed higher resolution *G*(*r*) correlations extending out past the traditional geometry, even after the correlations of the traditional geometry had decayed to near zero. The extended long-range correlations are resolved as a result of the inclined geometry, firstly by increasing the maximum value of *Q* and secondly as a result of the higher *Q*-space resolution in the low scattering angle regions, as implied by |∇*Q*(*x*_P_, *y*_P_)| in Figs. 4 ▸(*c*) and 4 ▸(*d*), allowing for the capture of higher frequency signals in *F*(*Q*) when Fourier transforming to *G*(*r*). Additionally, |∇*Q*(*x*_P_, *y*_P_)| in the inclined geometry decays in a more natural manner, beginning as higher *Q* resolution at lower scattering angles and decaying to lower *Q* resolution at higher scattering angles. The opposite trend is true for the traditional geometry. Capturing the high frequency correlations in the low scattering angle regions where the signal is most intense is of greater benefit to establishing the long-range order.

The physical design of a flat-panel area detector can vary greatly by manufacturer and model. Specifically, the thickness of the scintillator and the mounting design can have large effects on the magnitude of the parallax and topography distortions introduced by a detector. It has been shown by many published reports that it is necessary to correct these distortions in the traditional geometry in order to measure and analyze X-ray scattering patterns properly (Weiß *et al.*, 2012 ▸; Lüthi *et al.*, 2019 ▸; Marlton *et al.*, 2019 ▸; Wu *et al.*, 2002 ▸; Zaleski *et al.*, 1998 ▸). Equivalently, it was observed that implementing the PE-1621 in the inclined geometry similarly necessitated distortion corrections for proper measurement and analysis.

Flat-panel area detectors are often manufactured for medical applications and commonly repurposed for X-ray total scattering measurements. In these cases, the mounting design of the scintillator is intended for the detector to be in the traditional geometry. In the inclined geometry an increase in the magnitude of the topography distortion is observed due to insufficient scintillator anchoring points, causing non­uniform detector sag (Lüthi *et al.*, 2019 ▸; Burns, 2022 ▸). Additionally, the large angles of incidence introduced by the detector tilt increase the magnitude of the parallax distortions in the measured pattern (Marlton *et al.*, 2019 ▸; Wu *et al.*, 2002 ▸; Zaleski *et al.*, 1998 ▸; Burns, 2022 ▸).

**Figure 10:**
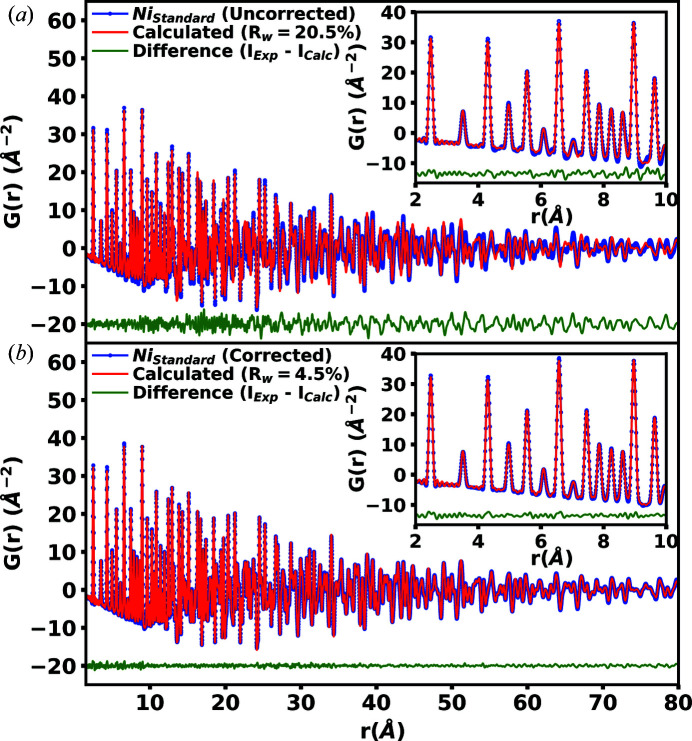
Comparison of powdered nickel pair distribution functions *G*(*r*) generated experimentally and modeled theoretically using the *PDFgui* software package (Farrow *et al.*, 2007 ▸), (*a*) before and (*b*) after the application of the parallax and topography corrections in the inclined geometry. Parallax and topography produce distortions in *G*(*r*) which result in a poor theoretical model fit of powdered nickel. After correction for the distortions, the theoretical fit is greatly improved.

In the inclined geometry, distortions from parallax and topography lead to more noticeable errors in peak positions and amplitudes in the calculated *G*(*r*) that need to be addressed. Using the X-ray total scattering pattern of a powdered silicon laboratory standard, the parallax and topography distortions were quantified and corrected. For detailed information on the parallax and topography distortion corrections used here, see the supporting information. Shown in Fig. 10 ▸ are the theoretical *G*(*r*) models generated by the *PDFgui* software package (Farrow *et al.*, 2007 ▸) for a powdered nickel sample (*a*) before and (*b*) after parallax and topography corrections.

**Figure 11:**
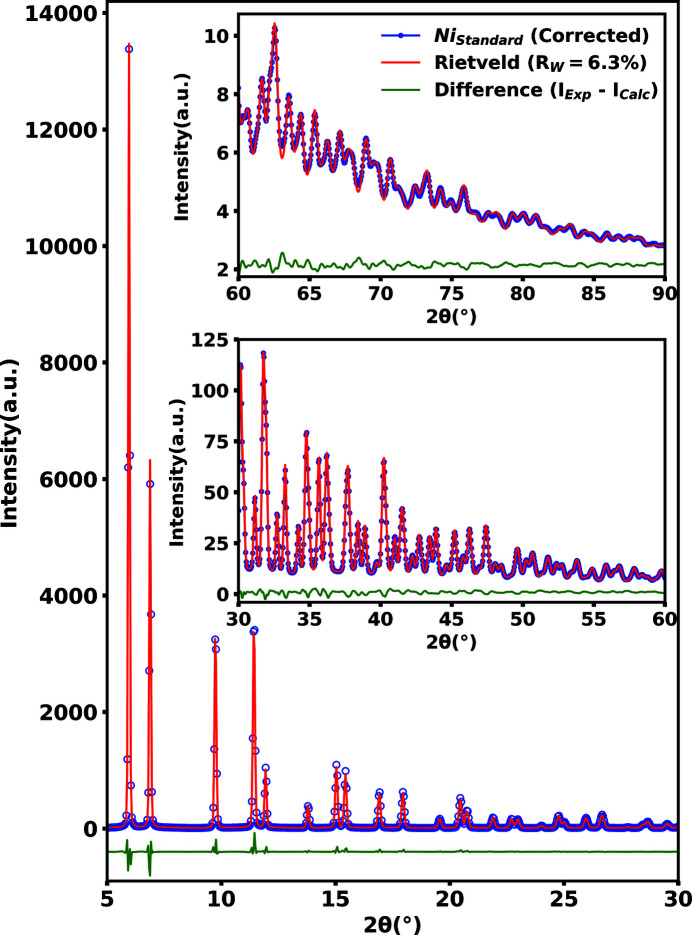
Rietveld refinement using the *GSAS-II* software package (Toby & Von Dreele, 2013 ▸) for powdered nickel after application of the parallax and topography correction in the inclined geometry.

The corrections are applied and any distortions in the pair distribution functions *G*(*r*) are greatly reduced, improving the peak positions and amplitudes. The quality of the fit is dramatically improved and a significant reduction is observed in the *R* factor *R*_W_ from 20.5 to 4.5%. The same distortions due to parallax and topography produce incorrect peak positions in the X-ray total scattering pattern. Fig. 11 ▸ shows the calculated Rietveld refinement generated by the *GSAS-II* software package (Toby & Von Dreele, 2013 ▸) for the powdered nickel after parallax and topography corrections. The Rietveld refinement produced an acceptable *R*_W_ of 6.3% over the entire selected scattering region out to 2θ = 90°. As shown in Figs. 10 ▸ and 11 ▸, with appropriate care taken to correct the parallax and topography distortions, the X-ray total scattering patterns in the inclined geometry can be corrected to levels adequate for both high resolution *G*(*r*) analysis and Rietveld refinements.

## 4. Conclusions

The inclined geometry produces distribution functions of improved quality compared with traditional geometries through two main mechanisms. First, the measurable scattering range is extended to 90°, increasing the accessible magnitudes of the reciprocal-space vector *Q* by 47%. Consequently, the generated reduced total scattering structure function *F*(*Q*) at an increased maximum *Q* decays to a lower intensity by means of the Debye–Waller factor. This reduces the magnitude of modification functions typically applied to *F*(*Q*) to satisfy the requirement of 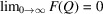 for the Fourier transform to *G*(*r*). In concert, by extending the measurable range in *Q* and reducing the extent of the applied modification functions to *F*(*Q*), the real-space resolution in the generated *G*(*r*) is preserved with minimal termination ripples. Secondly, the inclined detector geometry shifts the maximum solid angle ΔΩ(γ) and the magnitude of the reciprocal-space vector gradient |∇*Q*(*x*_P_, *y*_P_)| away from the beam center to the scattering angle 2θ = 90° − α along the vertical scattering plane. The shift results in the measured counts per pixel at the lower scattering angle regions being reduced, while increasing the measured counts per pixel at higher scattering angle regions, leveling the dynamic range of the measured total scattering pattern. Reducing the intensity of potentially saturating signals in the lower scattering angle region allows for the incident beam flux to be increased. In concert, these effects lead to a sixfold increase in coherent scattering signal-to-noise ratio compared with the traditional geometry, implying that a measurement of the same quality at the traditional geometry’s maximum value of *Q* could be acquired measuring 36 times fewer snapshots, greatly reducing acquisition time.

While distortions and artifacts are accentuated in the inclined geometry, these effects are well known in the traditional geometry (Weiß *et al.*, 2012 ▸; Lüthi *et al.*, 2019 ▸; Marlton *et al.*, 2019 ▸; Wu *et al.*, 2002 ▸; Zaleski *et al.*, 1998 ▸). Through careful distortion and gain corrections, the errors can be corrected to an unobserved level.

Comparing the two detector configurations, using an inclined geometry provides higher quality reciprocal-space information with better statistics. The inclined geometry enables the acquisition of high quality data in less time. It extends the measurable scattering range to 90°, increasing the accessible magnitudes of the reciprocal-space vector *Q*. The inclined geometry enables acquisition of higher quality data for simultaneous Rietveld refinement and total scattering studies, yielding structural information on the short-, medium- and long-range orders from one single measurement.

## 5. Related literature

For further literature related to the supporting information, see Kieffer *et al.* (2020 ▸), O’Donnell *et al.* (2018 ▸), Shah (1971 ▸) and Soper & Barney (2012 ▸).

## Supplementary Material

Mathematical background. DOI: 10.1107/S1600576723001747/xx5017sup1.pdf
