# Supplementary material for: An inclined detector geometry for improved X-ray total scattering measurements
Source: J Appl Crystallogr. 2023 Apr 1;56(Pt 2):510–8. doi: 10.1107/S1600576723001747 (PMC10077857; doi:10.1107/S1600576723001747)
Supplement: Supplementary file 1 [file j-56-00510-sup1.pdf]

JOURNAL OF APPLIED CRYSTALLOGRAPHY

Volume 56 (2023)

Supporting information for article:

An inclined detector geometry for improved X-ray total scattering measurements

Nicholas Burns, Aly Rahemtulla, Scott Annett, Beatriz Moreno and Stefan Kycia

SUPPLEMENTARY MATERIALS
An Inclined Detector Geometry for Improved X-ray Total Scattering Measurements

Nicholas Burns,[a] Aly Rahemtulla,[a,b] Scott Annett,[a] Beatriz Moreno[a,b] and Stefan Kycia [a]*

[a] *University of Guelph, Guelph, Ontario, Canada, and* [b] *Canadian Light Source, Saskatoon, Saskatchewan, Canada. E-mail: skycia@uoguelph.ca*

1. Distortion Corrections

The measured x-ray total scattering patterns collected with our PE-1621 detector in both the traditional and inclined configurations are found to be distorted in a manner that cannot be easily corrected for by changing the geometry. In order to quantify and correct the observed distortions, a methodology is presented.

The x-ray total scattering pattern of a powdered silicon lab standard (NIST SRM 640f) is measured in both detector configurations at a temperature of 100K. The detector geometry for each configuration is then calculated by scattering pattern refinement of experimentally measured silicon and a theoretically calculated $Fd\bar{3}m$ silicon calibrant. The silicon pattern is generated at a lattice constant of $5.42940\mathring{A}$, the reference value for $Fd\bar{3}m$ silicon at 100K, as presented by (Shah, 1971). The silicon pattern generation and geometry refinements are performed using the PyFAI python library (Kieffer *et al.*, 2020). Generation of the detector geometries allows for the assignment of the scattering angle $2\theta_{Geometry}$ and the azimuthal angle $\chi_{Geometry}$ to each detector pixel. Additionally, the magnitude of the reciprocal space vector $Q_{Geometry}$ can be

calculated for each pixel using Equation 1, where λ is the wavelength of the incident x-ray beam.

$$Q = \frac{4\pi}{\lambda} \sin\left(\frac{2\theta}{2}\right) \tag{1}$$

The geometry refinement process minimizes the difference between the theoretically calculated and experimentally measured Debye-Schrrer rings. The Debye-Schrrer rings, in both theoretical and experimental scattering patterns, span the full range of $\chi_{Geometry}$. A theoretical Debye-Schrrer ring is assigned a single $Q_{Geometry}$ value defined as $Q_{Calculated}$ across the entire range in $\chi_{Geometry}$. Conversely, the experimental Debye-Schrrer rings are measured at an array of assigned $Q_{Geometry}$ values defined as $Q_{Observed}$ across the entire range in $\chi_{Geometry}$. The geometry parameters are refined, redistributing the calculated $Q_{Geometry}$ for each pixel of the detector to minimize the differences between the assigned values of $Q_{Calculated}$ and $Q_{Observed}$ for each Debye-Schrrer ring. However, a flat panel area detector such as the PE-1621 contains innate distortions which produce differences between the assigned values of $Q_{Calculated}$ and $Q_{Observed}$, which cannot be corrected by changes in geometry.

A distortion correction is necessary to address the remaining deviations between $Q_{Calculated}$ and $Q_{Observed}$ that could not be corrected for by the geometry refinement. Quantifying the distortion correction entails dividing the two-dimensional x-ray total scattering pattern into sampled regular intervals spanning $2\theta_{Geometry}$ and $\chi_{Geometry}$. Each sampled interval consists of a wedged shape region with dimensions of 5° in $2\theta_{Geometry}$ by 3° in $\chi_{Geometry}$. The set of sampled intervals forms a grid across the surface of the detector containing 720 sampled regions in the traditional geometry and 540 sampled regions in the inclined geometry. Within each sampled region, the total scattering signal is interpolated to a regular two-dimensional grid with dimensions $Q_{Geometry}$ by $\chi_{Geometry}$ using custom Python code and then integrated along $\chi_{Geometry}$

to a one-dimensional pattern in $Q_{Geometry}$. The one-dimensional patterns assigned to each sampled interval are then all Rietveld refined individually using scriptable GSAS-II (O'Donnell *et al.*, 2018; Toby & Von Dreele, 2013).

In each Rietveld refinement, the wavelength of the incident x-ray beam λ is made a variable parameter while the lattice constant is set to a fixed value. Using Equation 1, the refined wavelength λ' gives the average $\overline{Q_{Observed}}$ in each sampled region. Once $\overline{Q_{Observed}}$ is calculated for all sampled regions, a spline fit enables $Q_{Observed}$ to be calculated for each detector pixel. $Q_{Observed}$ is then used when interpolating the measured scattering patterns to regular two-dimensional grids of dimensions $Q_{Observed}$ by $\chi_{Geometry}$. Using $Q_{Observed}$ in place of $Q_{Geometry}$ corrects the distortions in the resulting interpolated patterns. Presented in Figure 3 are the quantified corrections to the magnitude of the reciprocal space vector $\Delta Q_{Correction}$, calculated by taking the difference between $Q_{Observed}$ and $Q_{Geometry}$ in refined geometries for the traditional and inclined configurations.

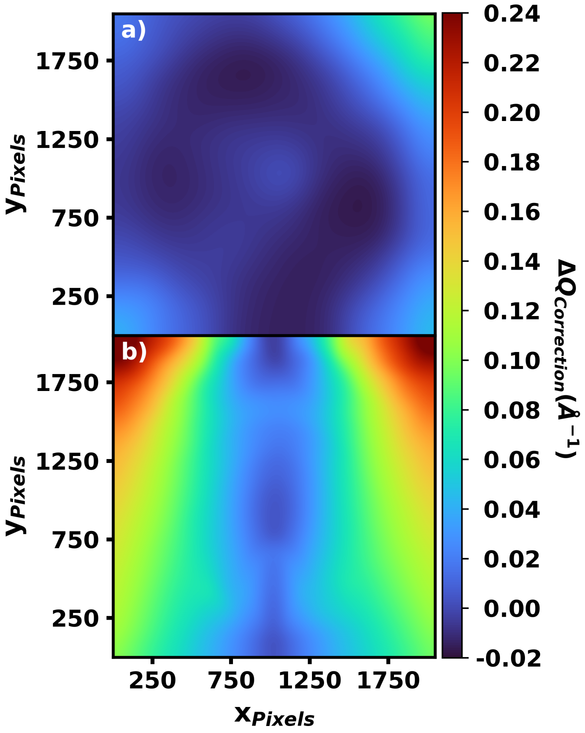

Fig. 1. Correction to the magnitude of the reciprocal space vector $\Delta Q_{Correction} = Q_{Observed} - Q_{Geometry}$ in a) traditional and b) inclined geometries. Each correction is calculated by quantifying the difference in Q for experimentally measured silicon and the theoretically calculated silicon calibrant at 100K.

2. PE-1621 Statistical Noise

The statistical noise of a single pixel in the PE-1621 detector was experimentally determined to follow Poisson-like statistics as defined in Equation 2.

$$\sigma_{Counts} = \sqrt{\beta N_{Counts}} \tag{2}$$

The statistics of a single pixel in the PE-1621 detector differs from Poisson statistics by a scaling factor β for a gain setting of 0.25pF (Michel *et al.*, 2006). In order to experimentally determine the statistical behavior of the detector a series of flood field measurements were conducted. The detector was uniformly illuminated with a series of intensities adjusted by increasing or decreasing the incident flux as seen by the

detector. A small region of interest (ROI) was selected where the intensity was most uniform and intense. At each selected incident flux, 1024 snapshots were collected. The standard deviation of the individual pixels σ_{Total} in the ROI for the 1024 snapshots was calculated using Equation 3. Here, I_i is the raw measured intensity of a pixel in a single snapshot and N is the total number of snapshots.

$$\sigma_{Total} = \sqrt{\frac{\sum_i^N (I_i - \overline{I})^2}{N-1}} \tag{3}$$

The behavior of the standard deviation of background $\sigma_{Background}$ is found to differ from the standard deviation of the measured counts σ_{Counts} and does not follow Equation 2. As a result the standard deviations add in quadrature (Hughes & Hase, 2010). Using the average σ_{Total} in the ROI, the standard deviation of measured counts σ_{Counts} can be determined. In order to extract the behavior of σ_{Counts} from σ_{Total} Equation 4 is used. Here, $\sigma_{Background}$ is the standard deviation of the background and $I_{Background}$ is the measured counts of the background. $\sigma_{Background}$ and $I_{Background}$ were experimentally determined to be an approximately uniform values of 25 and 4900 respectively.

$$\sigma_{Counts} = \sqrt{\sigma_{Total}^2 - \sigma_{Background}^2} \tag{4}$$

After calculating σ_{Counts} the Poisson-like function defined in Equation 2 is fit to the experimental values where a β value of 2.83 is determined.

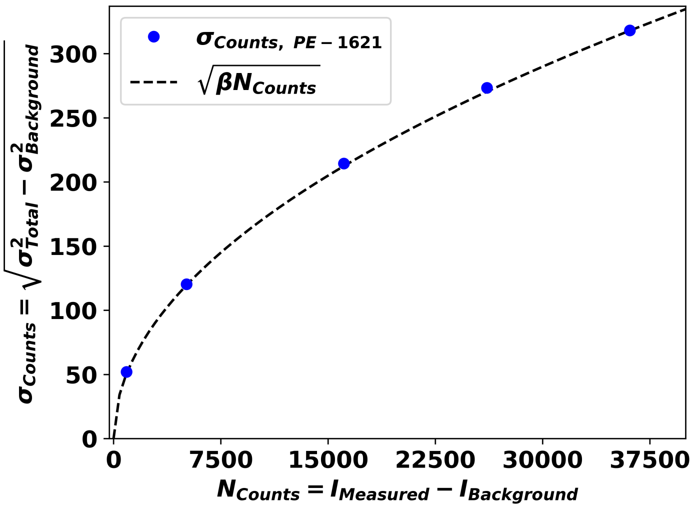

Fig. 2. Single pixel standard deviation of measured counts σ_{Counts} for the PE-1621 detector. The standard deviation of measured counts σ_{Counts} is found to follow Poisson-like statistics $\sigma_{Counts} = \sqrt{\beta N_{Counts}}$ where, $\beta = 2.83$. An experimentally determined background of 4900 counts is subtracted from all measured intensities. Additionally, an experimentally determined standard deviation of the background $\sigma_{Background}$ of 25 is subtracted from the all calculated σ_{Total} values in quadrature in order to calculate σ_{Counts}. The detector is set to a gain of 0.25pF for all measurements.

Similarly the statistical behavior of the ensemble can be explored. The ensemble consists of binning snapshots and pixels, each type of binning is investigated individually. The standard deviation of the ensemble $\sigma_{Mean\ Total}$ is similarly calculated in a ROI using Equation 3 after the binning has been performed. The average of $\sigma_{Mean\ Total}$ in the ROI is found to have the ensemble behavior defined in Equation 5 and is presented in Figures 3 and 4.

$$\sigma_{Mean\ Total} = \frac{\sigma_{Total}}{\sqrt{N_{Snapshots}}\sqrt{N_{Pixels}}} \tag{5}$$

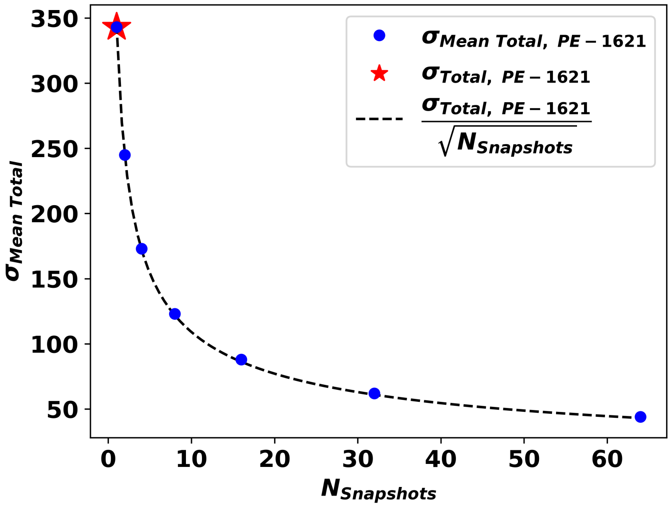

Fig. 3. Ensemble standard deviation for binned snapshots $\sigma_{Mean\ Total}$ using the PE-1621 detector. The standard deviation after snapshot binning is found to follow the ensemble behavior $\sigma_{Mean\ Total} = \frac{\sigma_{Total}}{\sqrt{N_{Snaphots}}}$. The detector is set to a gain of 0.25pF for all measurements.

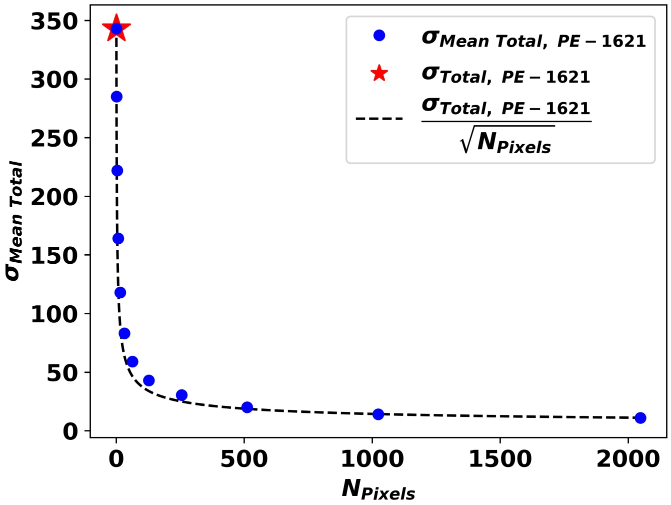

Fig. 4. Ensemble standard deviation for binned pixels $\sigma_{Mean\ Total}$ using the PE-1621 detector. The standard deviation after pixel binning is found to follow the ensemble behavior $\sigma_{Mean\ Total} = \frac{\sigma_{Total}}{\sqrt{N_{Pixels}}}$. The detector is set to a gain of 0.25pF for all measurements.

Further, the measured background signal is statistically independent of the background signal in the measured active image. As a result subtraction of the background

from the active image increases the standard deviation of the background $\sigma_{Background}$ in quadrature. This effect occurs for subtraction of air scattering and capillary signals from the active measurement. Due to the subtraction of these signals $\sigma_{Background}$ is experimentally determined to be approximately 53 for 1s exposures.

3. Modification Functions

The inclined geometry provides access to a broader range in Q. The additionally probed reciprocal-space helps to preserve real-space resolution by allowing the reduced total scattering structure function $F(Q)$ defined in Equation 6 to damped naturally due to the Debye-Waller factor.

$$F\left(Q\right) = Q\left[S\left(Q\right) - 1\right] \tag{6}$$

Natural dampening of the $F(Q)$ does not require the full extent of damping produced by modification functions M(Q) such as the Lorch function and modified versions (Lorch, 1969). Modification functions are applied to the reduced total scattering structure function by multiplication where $F'(Q) = F(Q)M(Q)$. The modification functions begin at unity and decay to zero at the maximum value of Q in order to reduce the extent of termination ripples appearing in the $G(r)$ (Soper & Barney, 2012). The modification functions aggressively dampen the envelope of the $F(Q)$, which is ideal for smaller maximum values of measured Q where the $F(Q)$ maintains significant signal. However, when patterns are measured out to a larger range in Q as in the inclined geometry, the $F(Q)$ decays to less amplitude and termination ripples are naturally reduced. As a result, the extent of damping applied by the modification functions can be decreased, while still achieving the same magnitude of termination ripples as generated in the traditional geometry. A modified version of the Lorch function is defined in Equation 7 in order to reach this effect. The Lorch function is modified by

taking the function to the power factor of ζ. From this modification, the decay from unity to zero is preserved over the Q-range. The extent of damping to the $F(Q)$'s envelope can be reduced by implementing values of $\zeta < 1$, preserving the natural real-space resolution.

$$M(Q) = \left[\sin\left(\frac{Q\pi}{Q_{max}}\right) \Big/ \left(\frac{Q\pi}{Q_{max}}\right)\right]^{\zeta} \tag{7}$$

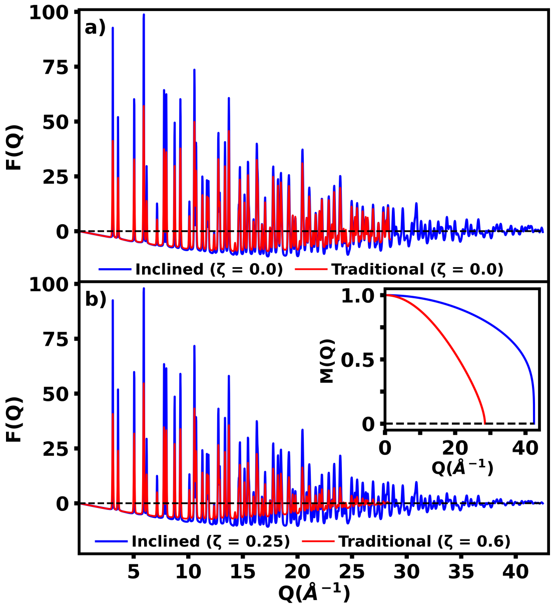

Fig. 5. Comparison of the reduced total scattering structure function $F(Q)$ in inclined and traditional geometries for powdered nickel using a) no modification function b) a Lorch power factor of $\zeta = 0.6$ in the traditional geometry and $\zeta = 0.25$ in the inclined geometry. The inset shows the modification functions $M(Q)$ applied to each geometry's $F(Q)$.

References

Hughes, I. & Hase, T. (2010). *Measurements and Their Uncertainties: A Practical Guide to Modern Error Analysis*, chap. 7.4, p. 95. OUP Oxford.

Kieffer, J., Valls, V., Blanc, N. & Hennig, C. (2020). *Journal of Synchrotron Radiation*, **27**(2), 558–566.

Lorch, E. (1969). *Journal of Physics C: Solid State Physics*, **2**(2), 229.

Michel, T., Anton, G., Böhnel, M., Durst, J., Firsching, M., Korn, A., Kreisler, B., Loehr, A., Nachtrab, F., Niederlöhner, D. *et al.* (2006). *Nuclear Instruments and Methods in Physics Research Section A: Accelerators, Spectrometers, Detectors and Associated Equipment*, **568**(2), 799–802.

O'Donnell, J. H., Von Dreele, R. B., Chan, M. K. Y. & Toby, B. H. (2018). *Journal of Applied Crystallography*, **51**(4), 1244–1250.

Shah, J. S. (1971). *Thermal lattice expansion of various types of solids*. PhD dissertation, University of Missouri–Rolla.

Soper, A. K. & Barney, E. R. (2012). *Journal of Applied Crystallography*, **45**(6), 1314–1317.

Toby, B. H. & Von Dreele, R. B. (2013). *Journal of Applied Crystallography*, **46**(2), 544–549.
